# The Role of Hydrogen Bonds in Interactions between [PdCl_4_]^2−^ Dianions in Crystal

**DOI:** 10.3390/molecules27072144

**Published:** 2022-03-26

**Authors:** Rafał Wysokiński, Wiktor Zierkiewicz, Mariusz Michalczyk, Thierry Maris, Steve Scheiner

**Affiliations:** 1 Faculty of Chemistry, Wrocław University of Science and Technology, Wybrzeże Wyspiańskiego 27, 50-370 Wrocław, Poland; 2 Département de Chimie, Université de Montréal, Montréal, QC H3C 3J7, Canada; thierry.maris@umontreal.ca; 3 Department of Chemistry and Biochemistry, Utah State University, Logan, UT 84322-0300, USA; steve.scheiner@usu.edu

**Keywords:** π-hole, counterions, molecular electrostatic potential, AIM, energy decomposition

## Abstract

[PdCl_4_]^2−^ dianions are oriented within a crystal in such a way that a Cl of one unit approaches the Pd of another from directly above. Quantum calculations find this interaction to be highly repulsive with a large positive interaction energy. The placement of neutral ligands in their vicinity reduces the repulsion, but the interaction remains highly endothermic. When the ligands acquire a unit positive charge, the electrostatic component and the full interaction energy become quite negative, signalling an exothermic association. Raising the charge on these counterions to +2 has little further stabilizing effect, and in fact reduces the electrostatic attraction. The ability of the counterions to promote the interaction is attributed in part to the H-bonds which they form with both dianions, acting as a sort of glue.

## 1. Introduction

More than a century of study of the hydrogen bond (HB) [1,2] has yielded a myriad of facts, ideas, and principles concerning this crucial linker in the microscopic world. It has been learned [3,4,5,6,7,8,9,10,11,12,13,14,15,16,17,18,19,20,21,22,23,24,25] that Coulombic forces are a critical factor, wherein the polarization of the R-H covalent bond induces a partial positive charge on the H which attracts an approaching nucleophile. This basic attraction is supplemented by a charge transfer from the lone electron pair of the nucleophile to the antibonding σ*(R-H) orbital of the acid. Other contributing factors arise from mutual polarization of the two subunits and London dispersion. The HBs that result from this confluence of phenomena are both ubiquitous and of enormous importance [7,12,14,26,27,28,29,30], essential for life, occurring within such biomolecules as proteins or nucleic acids, enzymatic reaction pathways, catalytic intermediates, and of course in water.

Of more recent interest are a number of other closely related noncovalent interactions, where the bridging H of the HB is replaced by any of a long list of atoms that lie on the right of the periodic table. These bonds are typically classified by the family of the bridging atom, e.g., halogen, chalcogen, tetrel, pnicogen, and triel bonds. However, they share with the HB many of the same contributing factors [31,32,33]. The bridging atom acquires a positive region, differing from the H only in that this region is more localized, which can similarly attract a nucleophile. Moreover, like the HB, these other noncovalent bonds are likewise stabilized by charge transfer, polarization, and dispersion [34,35,36,37,38,39,40,41,42].

As a ubiquitous and powerful force, the HB contributes heavily to assembling and preserving the architecture of supramolecular synthons [15,43,44,45,46,47,48,49,50,51,52,53,54,55]. Of the sorts of assemblies to which H-bonds contribute, among the most intriguing are those that contain “like–like charge” interactions where ions of like charge lie adjacent to one another. These anion···anion [56,57,58,59,60,61,62,63,64,65,66,67,68,69,70,71,72] and cation···cation [72,73,74,75,76,77] interactions are counterintuitive and have generated recent and extensive scrutiny [5,77,78,79,80,81], being called among other names an “anti-electrostatic” hydrogen bond (AEHB) [78,82]. In one picture, the Coulombic repulsion is overcome by resonance-type covalency represented by n→π*/n→σ* charge transfer [82]. Another view claims that the nominal point charge-point charge repulsion is oversimplified [83], and the full electrostatic term is more complicated, arising from the charge distribution over the entire subunit, as well as charge penetration effects. Another factor helping to overcome the implicit repulsion is the cooperativity of hydrogen bonding not only in simple dimers, but also within larger clusters [73], with a supporting role played by dispersion. Such cooperativity has been confirmed from both spectroscopic (IR, NMR) and computational perspectives [73].

Our own group has extended the understanding of this question to anion–anion interactions that involve π-holes [59,60,61,66,67,68,69,70,71]. These systems were stabilized by an assortment of noncovalent bonds, including pnicogen, triel, spodium, noble gas, and alkali earth bonds. The calculations showed that these complexes were metastable in the gas phase, wherein the dissociation was impeded by an energy barrier, but were fully stable in solution, despite their like charges. The innate attractive forces in systems such as tetrachloridopalladate(II) or trichloridomercurate(II) units) [66,68] were demonstrated by AIM, NBO, and NCI analyses, supported by experimental data. The results showed how the presence of counterions could stabilize these anion–anion interactions, in large part through the attenuation of the charges residing on the interacting anions.

One very recent study in particular [66] explored the interaction between a pair of [PdCl_4_]^2−^ dianions. The double charge on each makes for a particularly repulsive naked Coulombic repulsion. Indeed, in the absence of any surrounding environment, these two dianions strongly repel one another. However, the inclusion of a few of the surrounding counterions, along with the H-bonds which they form to these dianions, enables the entire system to be held together as seen in the crystal. In this case, the two dianions are held together in part by a charge transfer from the Cl lone pair of one unit to the vacant Pd π-orbitals lying above the plane of the other.

The present work is designed to explore the precise mechanism whereby this nominally highly dianion–dianion repulsion can be overcome by such external species. What is the relative importance of the charges on these surrounding molecules as compared to the H-bonds which they form with the anions? Is this a purely electrostatic phenomenon, or are there strong elements of polarization and charge dispersal which are important? Are there any specific stabilizing interactions between the pair of [PdCl_4_]^2−^ units which can act to hold them together if the overall Coulombic repulsion can be overcome, and how might these noncovalent bonds be affected by the surrounding molecules?

**Figure 1:**
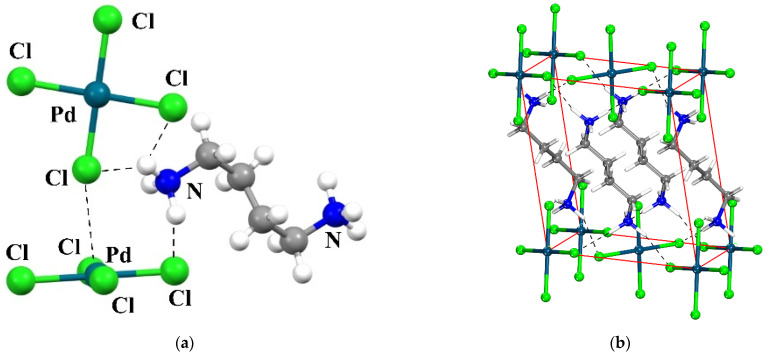
View of the crystal structure of the studied system (CSD REFCODE: NETMOO [84]). (**a**): View of the PdCl_4_^2−^ anions (**b**) View of one unit-cell content showing the layered character of the structure. In both views, the shortest hydrogen bonds are shown as dashed lines.

The analysis is designed to focus on a specific system whose crystal structure has been determined as an example. Figure 1 displays the relevant portion of the NETMOO [84] system, which shows some of the most important interactions. One can see the contact between the Cl of the upper unit and the Pd of that below. Quantum calculations attributed this arrangement to a π-hole bond wherein Cl lone pairs of one unit transfer charge to vacant orbitals above the Pd center of its neighbor [66]. It is also apparent that the NH groups of the counterion can engage in NH···Cl H-bonds with either of the dianions. As a starting point, the two [PdCl_4_]^2−^ anions are placed in the positions which they occupy in the crystal. Then, various models, of various size and complexity, of the counterions are added to the system in stages, monitoring the strength and nature of the interactions. The size of the counterion is examined by the comparison of the full ^+^NH_3_CH_2_CH_2_CH_2_CH_2_NH_3_^+^ species which occurs in the crystal with shorter versions such as Ca^2+^. Not only is the latter much smaller, but it is unable to engage in H-bonds. Other model ligands were considered of charge +1 and 0 so as to monitor the effect of the overall ligand charge. For example, removing a proton from ^+^NH_3_CH_2_CH_2_CH_2_CH_2_NH_3_^+^ yields the very similar but monocationic ^+^NH_3_CH_2_CH_2_CH_2_CH_2_NH_2_ whose effects can likewise be compared with the much smaller NH_4_^+^ and with K^+^ as a non-H-bonding cation. The models can be extended to those with no charge at all, such as NH_2_CH_2_CH_2_CH_2_CH_2_NH_2_, NH_3_, and Ar. Lastly, one can isolate the effects of a purely electrostatic treatment by replacing any of these species with a series of point charges, incapable of accepting any charge from any of the participating units, or engaging in any noncovalent bonding of any sort.

## 2. Computational Methods

Quantum calculations were carried out with the aid of the Gaussian 16, Rev. C.01 set of codes [85]. DFT computations employed the PBE0-D3 functional with the explicit inclusion of dispersion corrections, along with the def2TZVP [86,87,88] basis set. The extrema of the molecular electrostatic potentials (MEP) were measured on the 0.001 au isodensity surface using the MultiWFN program [89,90]. NBO analysis (NBO 7.0 [91]) probed the details of charge transfer and supplied natural atomic charges. Bader’s AIM methodology [92] elucidated bond paths and quantitative measures of their strength via the AIMAll suite of programs [93]. The decomposition of interaction energies was carried out at the PBE0-D3/ZORA/TZ2P level of theory through the ADF-EDA procedure according to the Morokuma–Ziegler scheme embedded in ADF software [94,95,96]. The solid-state geometries were accessed through the Cambridge Structural Database (CSD, ver. 5.42 with updates) and supporting CCDC software, Mercury and ConQuest [97,98]. Theoretical computations were based on the NETMOO [84] crystal structure. Heavy atoms were fixed in their crystal coordinates, and the H atom positions optimized. The basis set superposition error (BSSE) was corrected via the standard counterpoise procedure [99].

## 3. Results

**Figure 2:**
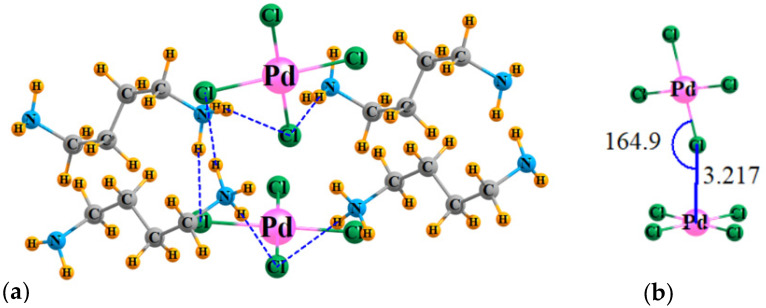
(**a**) Geometry of the PdCl_4_^2−^ dimer, surrounded by four counterions, with coordinates taken directly from X-ray structures; H atom positions optimized. (**b**) Detailed structure of [PdCl_4_^2−^]_2_. Distances in Å, angles in degs.

The geometry of the model system, taken directly from the X-ray coordinates, is exhibited in Figure 2a, which surrounds the PdCl_4_^2−^ dimer by four counterion ligands. The reader should be aware that a finite excerpt from a full crystal structure is considered here. There are a multitude of H-bonds connecting these counterions to the dianions. To avoid overcomplication of the figure, only very short ones, with R(H···Cl) less than 2.4 Å, are shown explicitly by the broken blue lines. Figure 2b focuses on the dianion dimer itself, showing clearly that the Cl of the top unit approaches the Pd of the lower to within 3.217 Å.

### 3.1. Direct Interactions between PdCl_4_ Units

The initial calculation focuses on the isolated PdCl_4_^2−^ dimer, in the absence of any counterions. The first row of Table 1 documents the strong repulsion between the two naked dianions. The interaction energy of the pair within their X-ray structure is +212 kcal/mol. Most of that can be attributed to a highly repulsive electrostatic component of +218 kcal/mol. Indeed, it would be difficult to generate any degree of attraction for the negatively charged Cl atom when the maximum of the MEP above the Pd atom is −371 kcal/mol, especially when coupled with the V_S,min_ on the Cl of −387 kcal/mol.

The next rows of Table 1 indicate the effects of adding four neutral ligands around this dianion pair. For the purposes of examining the interactions of the two principal dianions, two of these ligands were assigned to each PdCl_4_ unit to compose a [PdCl_4_]^2−^L_2_ subunit. The MEP was computed for each [PdCl_4_]^2^^−^L_2_ subunit, and the interaction energy between them was computed as the energy of the dimerization reaction (1)

2 [PdCl_4_]^2−^L_2_ → [PdCl_4_]^2−^_2_L_4_ (1)

The Ar atoms were placed at the positions of the proximate N atoms of the NH_3_-(CH_2_)_4_-NH_3_^2+^ counterions within the X-ray structure, as were the N atoms of the NH_3_ units. The H atoms of the latter were optimized and thus engaged in NH···Cl H-bonds with the anions. The Ar atoms have essentially no effect on the repulsive energy between the two anions, diminishing it by only 3 kcal/mol. The Ar atoms do reduce the negative value of V_S,max_, lowering its magnitude from −371 to −184 kcal/mol. They also strongly reduce the negative potential on Cl, lowering V_S,min_ from −387 to −202 kcal/mol. However, the electrostatic component of the interaction is little changed, dropping from +218 to +206 kcal/mol. Nor does Ar absorb any of the negative charge of these anions, leaving their total charge at −2.00.

The H-bonds connected with NH_3_ have a larger impact, albeit still fairly small. V_S,max_ drops a bit more, down to −172 kcal/mol, and V_S,min_ is reduced as well, causing a drop in E_ES_ to +173 kcal/mol. The NH_3_ units absorb a small amount of density, leaving the charges on the PdCl_4_ dianions at −1.96. Nevertheless, the interaction energy remains high at +182 kcal/mol. Extending the NH_3_ units to the full NH_2_(CH_2_)_4_NH_2_ ligands, likewise capable of engaging in NH···Cl H-bonds, has a further stabilizing effect. These longer species absorb a bit more of the anion’s charge, reducing it to −1.92, and raises V_S,max_ a small amount, up to −162 kcal/mol and also reducing the magnitude of V_S,min_. The electrostatic component and interaction energy are accordingly reduced as well, both down below +160 kcal/mol.

In order to distinguish the effects of this longer ligand arising from purely electrostatic considerations, from H-bonding, polarization, dispersion, and so on, these four NH_2_(CH_2_)_4_NH_2_ ligands were each replaced by a series of point charges. There was a one-to-one replacement of each atom of the ligand by such a charge, which was superimposed on the atomic position, and was assigned the natural charge equal to that of the ligand within the complex. The next row of Table 1 shows that this constellation of point charges has a small stabilizing effect on the dianion repulsion, less than that of the true ligand, and only roughly equivalent to the much smaller NH_3_ molecule. Of course, as simply a collection of charges, these pseudoligands cannot absorb any charge, so that of each ligand remains at −2.00. So, it is clear that the H-bonds connected with the full ligands, as well as any charge which they can accept from the anions, have a significant effect on stabilizing the anion pair, albeit far too small to make this interaction attractive.

A second iteration of this analysis would involve placing a positive charge on each ligand. The simplest such counterion, and one incapable of engaging in a H-bond, would be a monatomic cation such as K^+^. As exhibited in the next row of Table 1, the inclusion of four such cations makes the interaction exothermic with negative values of E_int_. The presence of these cations also strongly reduces the negative values of both V_S,max_ and V_S,min_, both smaller in magnitude than −80 kcal/mol. These changes are partly responsible for the negative, attractive electrostatic component at −94 kcal/mol.

The E_int_ of −97 kcal/mol is enhanced to −111 kcal/mol if the K^+^ is morphed into the NH_4_^+^ ion of roughly the same size, but with the added capability of forming NH···Cl H-bonds. This mutation has a small reducing effect on the MEP extrema and drops the formal charge on the PdCl_4_ unit to −1.80, adding to a slightly more negative E_ES_, which contributes to the more negative interaction energy. Enlarging the ammoniums to the much longer NH_2_(CH_2_)_4_NH_3_^+^ was done in such a way that it is the positively charged NH_3_^+^ unit that is placed close to the PdCl_4_ species. This counterion enlargement has a slightly deleterious effect, increasing E_int_ from −111 to −101 kcal/mol, as well as dropping the electrostatic attraction energy by 5 kcal/mol. This rise in E_int_ may be due to the lesser concentration of the positive charge in the larger cation. Each of these counterions, whether NH_4_^+^ or NH_2_(CH_2_)_4_NH_3_^+^, involves itself in two NH··Cl H-bonds for a total of four such bonds with each PdCl_4_ unit. The replacement of the ligand atoms by their corresponding point charges is just slightly less effective, with E_int_ becoming less exothermic by 3 kcal/mol.

It may be noted further from Table 1 that the electrostatic components are all quite attractive when any of these monocations are added, nearly −100 kcal/mol. On the other hand, even with these counterions present, V_S,max_ on Pd remains negative by between 53 and 58 kcal/mol. This contradiction argues against taking a positive V_S,max_ as a condition for an exothermic association. In sum, adding a +1 charge to the surrounding ligands enables them to absorb a bit more of the (PdCl_4_)^2−^ dianion’s negative charge. Most effective in this regard is the set of four ammonium cations which drop the dianion’s charge down to −1.80.

The last few rows of Table 1 refer to the addition of two dications instead of the four monocations. (The smaller number of the former is necessary in order to maintain overall electroneutrality. Equation (1) must be modified to describe each subunit as [PdCl_4_]^2−^L^2+^). Despite the reduction in their number, the dications prove more effective at promoting a more exothermic association. Whether the small monatomic Ca^2+^ or the much larger NH_3_(CH_2_)_4_NH_3_^2+^ dications, E_int_ drops below −120 kcal/mol. Even the collection of point charges designed to mimic the long ligand dication is effective in this regard, with E_int_ of –108 kcal/mol. The comparison of the Ca^2+^ and the L^2+^ systems enables some assessment of H-bonds, which are only possible for the latter. It is intriguing that although the H-bonding ligands leave both V_S,max_ and V_S,min_ more negative, the total electrostatic term is nonetheless more attractive when compared to Ca^2+^.

In fact, upon moving the analysis to the dications, the electrostatic component diverges substantially from the full E_int_. These two quantities differ by some 60–80 kcal/mol. More specifically, while the transition from monatomic K^+^ monocation to Ca^2+^ dication makes E_int_ more negative, and the same sort of change can be seen from L^+^ to L^2+^, it has the opposite effect on E_ES_, which becomes substantially less negative. This change to a less attractive electrostatic term contrasts with the less negative V_S,max_ quantities associated with the monatomic ions. Despite their smaller number, the Ca^2+^ dications are much better at dispersing the negative charge on the central dianions than K^+^, dropping this charge down to −1.73. There is much less distinction between the longer ligands, where the dications are slightly poorer at absorbing this charge than the monocations.

### 3.2. Secondary Interactions

It must be understood that the interaction energy between the two subunits is not wholly due to the Pd···Cl bond. The electrostatic term arises from interactions between the entire charge distributions of each subunit, which includes not only the central PdCl_4_ but also any ligands appended to it. There are also polarization and dispersion energies that involve the entire electron clouds. Added to that are a number of specific noncovalent contacts as well. For example, the NH groups on the small NH_3_ and NH_4_^+^ entities on one subunit can engage in H-bonds with the Cl atoms of the PdCl_4_ of the other, but also NH··N H-bonds with one another. The same is true of the larger ligands comprised of NH and CH proton donors. Even the monatomic counterions, such as K^+^ and Ca^2+^, are capable of forming specific bonding contacts with the Cl atoms of the opposite subunit.

**Figure 3:**
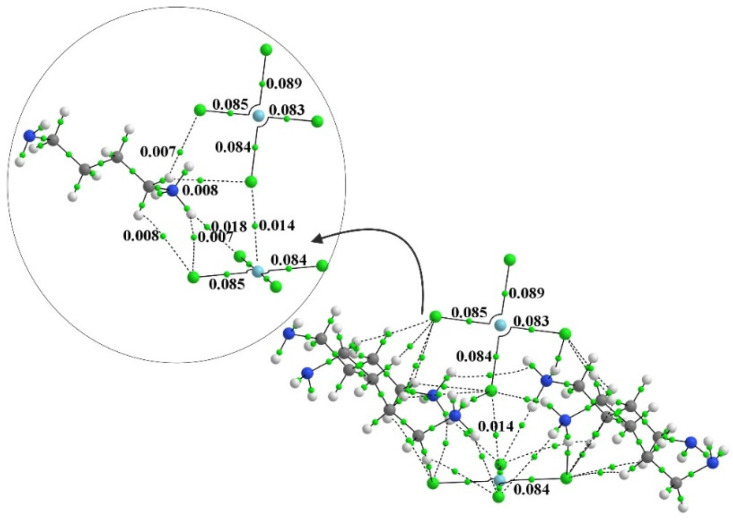
The AIM diagram of [NH_3_-(CH_2_)_4_-NH_2_]_4_[PdCl_4_]_2_. Numbers refer to bond path critical point in au.

**Figure 4:**
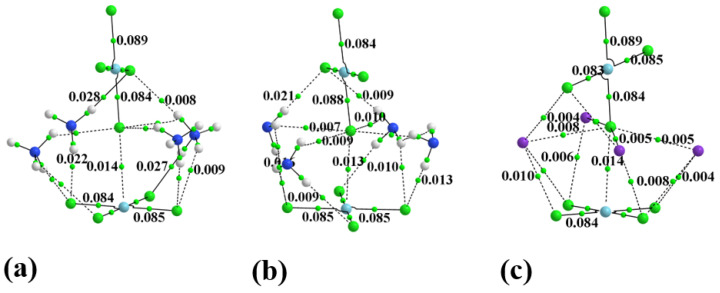
The AIM diagram of dianion surrounded by (**a**) 4 NH_4_^+^, (**b**) 4 NH_3_, and (**c**) 4 K^+^.

One can elucidate such interactions via the examination of AIM bond paths. In order to convey some sense of the number of these bonds, the AIM diagram of the system containing four full monocationic ligands is provided in Figure 3. There is a multitude of H-bonds and other noncovalent interactions between these ligands and both PdCl_4_ units, and even between one another. The inset to Figure 3 focuses on the dianion pair and one of these ligands for greater clarity. Figure 4 places clearly in evidence the various H-bonds that arise when these ligands are replaced by the smaller NH_4_^+^, NH_3_, and K^+^ species. The chief markers of the strengths of these various interactions are contained in Table 2. The first column displays the density of the Pd···Cl bond path between the two subunits, which seems to be relatively constant at 0.013 au. This nearly fixed amount is not surprising in view of the fact that the intermolecular Pd···Cl distance was held constant at its X-ray value regardless of the addition of any ligands, and ρ_BCP_ has been shown repeatedly in the literature [12,100,101] to be very sensitive to this interatomic distance. Prior works in the literature [102] have found and used a relationship that ties the energy of a noncovalent bond to ½ V, where V represents the potential energy density at the bond critical point. The use of this relationship leads to an estimate of the Pd··Cl bond energy as roughly 3 kcal/mol, as listed in the penultimate column of Table 2.

The other two columns of Table 2 report the bond critical point density and potential energy density as a sum of all bond paths that stretch between the two subunits, exclusive of Pd···Cl. These values suggest that the Pd···Cl bond is only part of the story, and that in a number of cases, AIM would suggest that the sum of the other noncovalent interactions exceeds this primary component. This energy sum from the last column of Table 2 shows a clear progression from monatomic species, such as Ar and K^+^, to the small H-bonding NH_3_ and NH_4_^+^, up to the largest ligands containing the connecting butyl chain. Notice also that this auxiliary sum drops off for the dicationic species.

Given the magnitude of the collective auxiliary noncovalent bonds within these complexes, it is perhaps not surprising that neither the total interaction energy nor even the full electrostatic term in Table 1 can always be closely related to the magnitudes of the MEP extrema on the Pd and Cl atoms, which concern only one of several noncovalent bonds. The ability of the counterions within the crystal structure to stabilize the entire lattice is mainly due to their effects on the PdCl_4_ units. Moreover, these ligands also act as a glue between PdCl_4_ units by forming H-bonds with both. This glue is further augmented by H-bonds between the counterions themselves.

As the electrostatic is the leading force stabilizing the current crystal, it is worth comparing the bonding between adjacent, oppositely charged atoms here with that which occurs within a common salt such as NaCl. The atoms of the [PdCl_4_]^2−^ dianions that come closest to one another are the Cl of one unit and the Pd of the other. This R(Pd···Cl) distance is 3.217 Å in the system described above, which is considerably shorter than 3.97 Å which corresponds to the sum of Pd and Cl vdW radii [103]. In NaCl, the R(Na···Cl) distance is only 2.8 Å in the crystal, also smaller than their vdW radii sum of 4.3 Å, so in this sense NaCl offers local attractive behavior that is parallel to that in the system under investigation here. If one now extracts a structure similar to the crystallographic arrangement of two [PdCl_4_]^2−^ from the NaCl lattice, i.e., a [NaCl_4_]^3−^···[NaCl_4_]^3−^ arrangement on lattice sites, it will become repulsive between the two units, just like in the Pd case. Adding neutral solvents will not cure this, but adding counterions will. So, qualitatively, a simple salt crystal will behave similarly to the Pd dianion system upon increasing the fragments investigated. Of course, the covalency of the PdCl bonds in the dianions along with the presence of the hydrogen bonds make a difference, but only on a quantitative level, which is explored in this work.

## 4. Conclusions

The interaction between the two naked PdCl_4_^2−^ dianions is clearly highly repulsive. The introduction of neutral ligands reduces the magnitudes of the negative MEP maxima and minima, which helps lower the electrostatic repulsion energy, but the interaction energy remains quite positive nonetheless, roughly equal to its total electrostatic component. Adding a positive charge to the ligands further reduces the magnitudes of the MEP extrema, although they remain negative. Nevertheless, these cations reverse the sign of the electrostatic and interaction energies, turning the latter exothermic by roughly 100 kcal/mol. Changing from four monocationic ligands to two dications reduces the total electrostatic attractive component but makes the total interaction energy a bit more exothermic. The stabilizing effect of the counterions is only partly due to the dispersal of the negative charges on the PdCl_4_^2−^ units or the reduction of the negative value of the π-hole on Pd. In a more global sense, the addition of the cationic ligands changes the formal charge on the entire subunit from −2 for naked PdCl_4_^2−^ unit to 0 after their introduction. The ability of these counterions to engage in H-bonds with both PdCl_4_ units further acts as a glue holding them together. A partial contribution to this structure cohesion is achieved via NH···Cl hydrogen bonds.

## Figures and Tables

**Table 1:** Interaction energy and its electrostatic component for interactions between subunits, and the maximum and minimum of the MEP of the uncomplexed subunits (kcal/mol), and total charge (Q, e) assigned to PdCl_4_ segment within complexes.

	E_int_	E_ES_	V_s,max_	V_S,min_	Q ^c^
(PdCl_4_)^2^^−^_2_	+212	+218	−371	−387	−2.00
neutral					
+4 Ar	+209	+206	−184	−202	−2.00
+4 NH_3_	+182	+173	−172	−194	−1.96
+4 L^0,a^	+157	+159	−162	−185	−1.92
+4(PC)^0,b^	+182				−2.00
+1					
+4 K^+^	−97	−94	−58	−75	−1.91
+4 NH_4_^+^	−111	−99	−56	−72	−1.80
+4 L^+^	−101	−94	−53	−70	−1.82
+4 (PC)^+^	−98				−2.00
+2					
+2 Ca^2+^	−121	−44	−27	−63	−1.73
+2 L^2+^	−124	−64	−64	−84	−1.85
+2 (PC)^2+^	−108				−2.00

^a^ L refers to the butyl ligands with amino groups on both ends. L^0^ has NH_2_ on both ends, L^+^ has NH_3_^+^ on one end near the PdCl_4_, L^2+^ has NH_3_^+^ on both ends for total charge of +2. ^b^ PC refers to the constellation of point charges that approximate L^0^, L^+^, or L^2+^, respectively. ^c^ total charge on each PdCl_4_ unit (average of two).

**Table 2:** AIM properties of bond critical points between subunits in complexes.

	ρ, au		−½ V, kcal/mol	
	Pd···Cl	Σothers ^a^	Pd···Cl	Σothers ^a^
(PdCl_4_)^2−^_2_	0.014	-	2.67	-
neutral				
+4 Ar	0.013	0.027	2.73	4.81
+4 NH_3_	0.013	0.053	2.77	8.93
+4 L^0,a^	0.013	0.058	2.80	10.03
+4(PC)^0,b^	0.013	-	2.74	-
+1				
+4 K^+^	0.014	0.028	3.00	4.54
+4 NH_4_^+^	0.014	0.066	3.01	10.79
+4 L^+^	0.014	0.071	3.01	11.60
+4 (PC)^+^	0.014	-	2.97	-
+2				
+2 Ca^2+^	0.014	0.012	2.89	1.69
+2 L^2+^	0.014	0.033	2.84	5.90
+2 (PC)^2+^	0.013	-	2.81	-

^a^ between [PdCl_4_]^2−^L_n_ units, *n* = 2 for neutral and monocationic ligands, 1 for dications. ^b^ PC refers to the constellation of point charges that approximate L^0^, L^+^, or L^2+^, respectively.

## Data Availability

The data presented in this study are available on request from the corresponding authors.

## Sample Availability

Samples of the compounds are not available from the authors.
